# Combined analysis of whole-exome sequencing and RNA sequencing in type 2 diabetes mellitus patients with thirst and fatigue

**DOI:** 10.1186/s13098-022-00884-z

**Published:** 2022-08-08

**Authors:** Bohan Lv, Xiuyan Yang, Tian An, Yanxiang Wu, Zhongchen He, Bowu Li, Yijiao Wang, Fang Tan, Tingye Wang, Jiajian Zhu, Yuanyuan Hu, Xiaokun Liu, Guangjian Jiang

**Affiliations:** 1 Traditional Chinese Medicine School, Beijing University of Chinese Medicine, Beijing, China; 2 Department of Endocrinology, Beijing He Ping li Hospital, Beijing, China; 3 Department of Cardiology, Gongren Hospital of Tangshan City, Tangshan, China

**Keywords:** Diabetes, Whole-exome sequencing, RNA sequencing, Fatigue, Mitochondria

## Abstract

**Background:**

The principal objective of this study was to gain a better understanding of the mechanisms of type 2 diabetes mellitus (T2DM) patients with fatigue (D-T2DM) through exome and transcriptome sequencing.

**Methods:**

After whole-exome sequencing on peripheral blood of 6 D-T2DM patients, the consensus mutations were screen out and analyzed by a series of bioinformatics analyses. Then, we combined whole-exome sequencing and transcriptome sequencing results to find the important genes that changed at both the DNA and RNA levels.

**Results:**

The results showed that a total of 265,393 mutation sites were found in D-T2DM patients compared with normal individuals, 235 of which were consensus mutations shared with D-T2DM patients. These genes significantly enriched in HIF-1 signaling pathway and sphingolipid signaling pathway. At the RNA level, a total of 375 genes were identified to be differentially expressed. After the DNA-RNA joint analysis, eight genes were screened that changed at both DNA and RNA levels. Among these genes, FUS and LMNA were related to carbohydrate metabolism, energy metabolism, and mitochondrial function. Subsequently, we predicted the herbs, including Qin Pi and Hei Zhi Ma, that might play a therapeutic role in D-T2DM through the SymMap database.

**Conclusion:**

These findings have significant implications for understanding the mechanisms of D-T2DM and provide potential targets for D-T2DM diagnosis and treatment.

**Supplementary Information:**

The online version contains supplementary material available at 10.1186/s13098-022-00884-z.

## Background

Diabetes is a severe worldwide problem threatening the health of millions of people. The number of individuals with type 2 diabetes mellitus (T2DM) is rapidly increasing worldwide. Current estimates indicate that by 2040, approximately 642 million people worldwide will be suffered from T2DM [1]. The occurrence of T2DM has been associated with genetic and acquired factors [2]. In Traditional Chinese Medicine (TCM), there are several different types of diabetes. One type of diabetes is called “Dual Deficiency of Qi and Yin Syndrome”, a common type of diabetes [3]. The main symptoms of this type are dry throat and mouth, fatigued spirit, and lack of strength. In modern medicine, some T2DM patients are also accompanied by thirst and frailty [4–8]. Hence, the diagnosis and treatment of this type of T2DM (D-T2DM) will become a problem facing the world.

Nowadays, with the emergence of next-generation sequencing technology, we can overcome the limitations of traditional genetic disease research methods. Whole-exome sequencing technology (WES) is a method that performs high-throughput analysis of all exon regions to sensitively identify rare and low-frequency disease-related mutations [9, 10]. WES involves three main steps to find the pathogenic genes: capture and enrichment of exome, high-throughput sequencing, and bioinformatic analysis. Several previous studies used WES to uncover variation associated with complex human traits. Some trials have explored the association between T2DM and exonic variants through WES, such as PAX4, RREB1, and PPP1R3A [11–13]. So far, however, there has been no detailed investigation of the changes in the exome of D-T2DM patients.

Therefore, this present study using the WES to find the specific mutation sites in D-T2DM patients compared with normal people. In addition, we performed functional enrichment and pathway analysis to explore the potential functions of the mutation gene. Finally, we combined exome and RNA sequencing to explore the pathogenesis of D-T2DM. The results may provide references for clinical application and introduce a new strategy for the treatment of D-T2DM.

## Methods

### Sample information description

Ethical approval accorded with the Declaration of Helsinki for this study was obtained from the Beijing University of Chinese Medicine ethics committee and Beijing Hepingli Hospital ethics committee. The primary inclusion and exclusion criteria are listed in Table 1. Finally, a total of 12 individuals, including six D-T2DM patients and six healthy subjects, were included in this study and provided informed consent. These individuals were separated into the D-T2DM group (patient ID: QYD001, QYD002, QYD003, QYD004, QYD005, and QYD006) and the control group (patient ID: WZC001, WZC002, WZC003, WZC004, WZC005, and WZC006). Then, fasting peripheral blood was collected from the participants and stored at – 80 ℃ for subsequent analysis.

**Table 1:** Inclusion and exclusion criteria

	Inclusion criteria	Exclusion criteria
Subjects with D-T2DM	Diagnosed with T2DM Diagnosed T2DM for at least 3 months Diagnosed with “Dual Deficiency of Qi and Yin Syndrome” according to TCM pattern diagnoses, including the key symptoms: dry throat and mouth, fatigued spirit and lack of strength	Diagnosed with type 1 diabetes, secondary diabetes, gestational diabetes, or unknown type of diabetes Patients with stage III hypertension or myocardial infarction Patients with severe primary diseases Patients with serious complications, such as infection and diabetic ketoacidosis
Healthy Subjects	FPG < 5.6 mmol/L Healthy and no associated symptoms of “Dual Deficiency of Qi nd Yin Syndrome”	Subjects with a family history of diabetes Subjects with hypertension or other cardiovascular and cerebrovascular diseases Subjects are currently taking medications

### DNA extraction and sequencing

After DNA was extracted, Agarose gel electrophoresis was used to check the quality of DNA, and Qubit 3.0 was used to determine the concentration of DNA. Next, in brief, DNA samples were fragmented by the Covaris instrument, then the sequencing library was constructed by end repair, A-tailing, and adapter ligation. After that, exome DNA was captured by Agilent SureSelect Human All Exon V6 Kit (Agilent). Then, the DNA libraries were attained after purification and PCR amplification. Finally, pair-end 150 bp sequencing was done using the Illumina sequencing platform.

### Bioinformatics analysis

Raw DNA sequencing data were aligned to the GRCh37/hg19, used as the reference genome for sequence alignment and subsequent analysis. The bioinformatics analysis involved quality assessment of sequencing data, mutation detection, mutation screening, and disease-related prediction.

#### Screening of mutation site

The mutation site screening was performed to analyze the single nucleotide polymorphisms (SNP) or insertion and deletion (InDel) information. Firstly, remove the mutation site with the reported frequency of > 1% by comparison with 1000h_all, esp6500si_all, gnomAD_ALL, and gnomAD_EAS database. Secondly, preserve the mutation of coding regions and spice sites. Thirdly, remove synonymous mutation and non-frameshift InDel mutation. Finally, retain the mutations that were harmful variations or cause splicing defects according to the SIFT, Polyphen, MutationTaster, and CADD software.

#### Mutation site harmful classification

According to the American Society of Medical Genetics and Genomics (ACMG) variant interpretation guidlines [14], the pathogenicity of mutations are classified into the categories as follows: pathogenic, likely pathogenic, uncertain significance, like benign and benign. Next, the number of mutation sites in each category was counted.

#### Analysis of the harmfulness of copy number variations (CNV)

CNVs are found in the biological genome, and some malignant CNVs can cause various diseases, including neurological disorders and cancer. CNVs were detected and annotated by CoNIFER and ANNOVAR software, respectively. After that, we further annotated the CNV results database using multiple databases, including DGV and CNVD database, to filter out benign CNVs and reserve malignant CNVs. Then, CNVs were classified into four types, including H (high), P (possibly deleterious), M (medium), and L (low).

#### Advanced analysis and DNA-RNA conjoint analyses

Further advanced analysis was conducted to identify the true mutations responsible for the disease from all mutation results. The biological function and involving pathway of mutation genes were enriched by Gene Ontology (GO) and Kyoto encyclopedia of genes and genomes (KEGG) pathway analysis. In addition, we collected disease-associated genes from the DisGeNet database and constructed the gene-disease correlation map. Then, protein interaction networks of the mutant genes were constructed using the gene network prediction tool GeneMania (http://genemania.org/). Finally, we screened significantly differentially expressed genes among the shared mutation genes and performed functional enrichment analysis with GO and KEGG pathway categories.

### Herb-gene interaction network

To predict herbs associated with the DE shared mutations, an herb-gene interaction network was constructed base on the SymMap database (https://www.symmap.org/). Predicted candidate herbs for each gene were presented and visualized with Cytoscape 3.8.2.

### Statistical analysis

All statistical analyses were accomplished by Student's independent-samples t-test using the SPSS software (Version 20.0). Results were expressed as mean ± SEM, and with P < 0.05 considered statistically significant.

## Results

### Participants information description

**Fig. 1:**
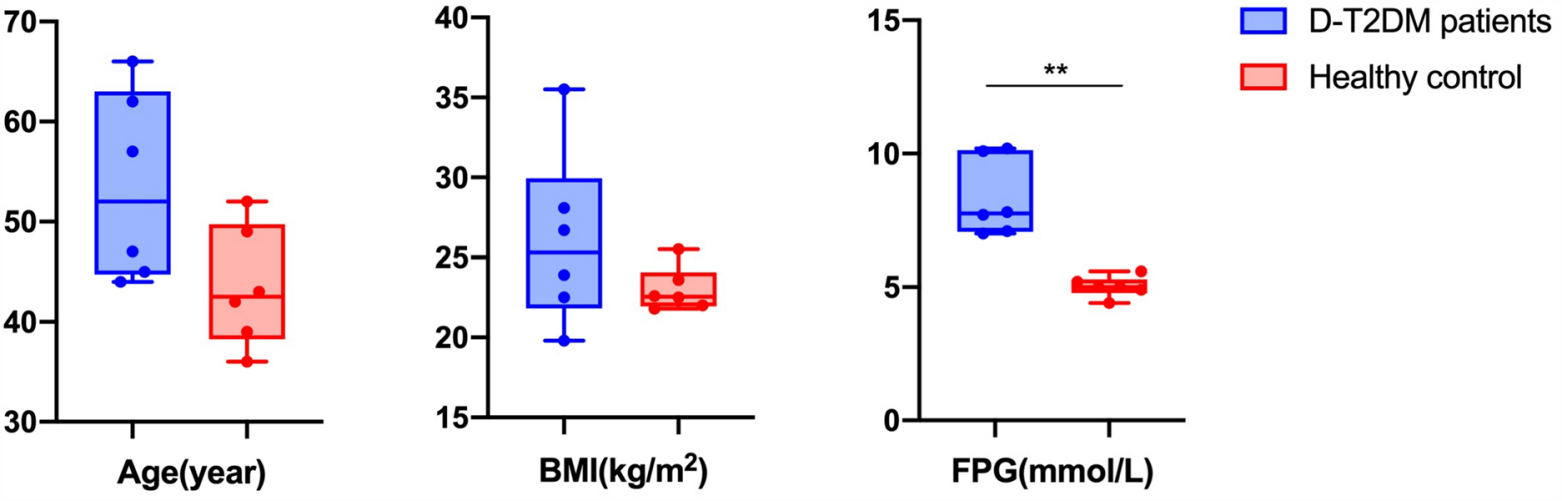
Characteristics of study subjects

At the beginning of the study, we recruited six D-T2DM patients (three males and three females) and six normal individuals (six females). All the patients fulfilled diagnostic criteria for D-T2DM. Figure 1 shows the characteristics of all individuals. There were no apparent differences in age and BMI between the two groups (P > 0.05). In addition, FPG levels were higher in the D-T2DM group when compared to the control group (P < 0.01).

### Whole-exome sequencing data summary

Whole-exome sequencing was performed on fasting peripheral blood of six D-T2DM patients and six normal individuals. Approximately 153.4 Gb of raw data with a 0.1% average error rate were obtained, and about 91.63% of raw reads had Qphred quality scores of > 30 (Additional file 1: Table S1). The clean reads were aligned and sorted to reference genome GRCh37/hg19 using BWA tools and SAMtools. 99.85% of the mapped content and 97.5% of the target fraction were covered with at least 10 × in depth. The average depth of sequencing was 124.96 (Additional file 1: Table S2).

### The results of variation detection

After obtaining mapped data, SAMtools was used to detect and filter the single nucleotide variant (SNV) sites. We detect a total of 265,393 exonic SNVs with 123,095 missense SNV, 886 stop-gain, and 94 stop-less. These SNVs lead to changes in amino acids during translation. Next, ANNOVAR software was used to annotate the SNPs. The results of annotation included the location, type, and conservative prediction of SNPs. Insertion and deletion (InDel) in the coding region and splice site can also cause acid changes during translation. Thus, we determined the count of InDel with different types on the coding region and the genome (Additional file 1: Tables S3 and S4).

### Screening and classification of the mutation sites

After fundamental analysis, the SNP/InDel information was screened for mutation sites, and finally, 3720 mutation sites were obtained (Table 2). According to ACMG's evidence, the mutation sites were classified into pathogenic, likely pathogenic, VUS, likely benign, and benign categories (Table 3). Lastly, a harmfulness analysis of CNV was performed and found a total of 160 malignant CNVs (Additional file 1: Table S5).

**Table 2:** The results of mutation sites screening (partial)

Priority	POS	ID	Gene Name	Exonic Func	Gencode
H	979560	rs762554040	AGRN	missense SNV	ENSG00000188157.14, ENST00000379370.6, ENST00000620552.4
H	1221564	rs61740392	SCNN1D	missense SNV	ENST00000379116.9, ENST00000325425.12, ENST00000400928.7, ENST00000379101.8, ENSG00000162572.20, ENST00000338555.6
H	1233779	rs544359869	ACAP3	missense SNV	ENST00000354700.9, ENST00000476572.1, ENST00000467278.5, ENST00000492936.5, ENSG00000131584.18, ENST00000353662.4
H	1262875	rs564546199	CPTP	missense SNV	ENST00000343938.8, ENST00000464957.1, ENSG00000224051.6
H	1269024		TAS1R3	missense SNV	ENST00000339381.5, ENSG00000169962.4
H	1269399	rs571862161	TAS1R3	missense SNV	ENST00000339381.5, ENSG00000169962.4
H	1269623	rs199779671	TAS1R3	missense SNV	ENST00000339381.5, ENSG00000169962.4
H	1309567	rs199844974	AURKAIP1	missense SNV	ENST00000378853.3, ENST00000338338.9, ENST00000321751.9, ENST00000338370.7, ENSG00000175756.13
H	1309675	rs758605382	AURKAIP1	missense SNV	ENST00000489799.1, ENST00000378853.3, ENST00000338338.9, ENST00000321751.9, ENST00000338370.7, ENSG00000175756.13

**Table 3:** Harmful classification screening results of each patient

Patient	Total	Pathogenic	LikelyPathogenic	VUS	LikelyBenign	Benign
QYD1	25,518	3	5	1240	1287	22,983
QYD2	25,540	4	6	1253	1264	23,013
QYD3	25,418	3	3	1262	1282	22,868
QYD4	25,398	1	7	1312	1226	22,852
QYD5	25,352	5	6	1241	1262	22,838
QYD6	25,897	3	4	1323	1244	23,323

### Screening of shared mutated sites between samples

After filtering the harmful mutation sites, the mutated genes shared by more than two patients were screened. At the same time, follow the principle that 90% of normal individuals did not share. We think the more patients have the same mutated gene, the more likely this mutation is to be associated with D-T2DM. The results indicated that there were 235 shared mutated sites in D-T2DM patients, and the top 15 were listed in Table 4. Among the results, the mutation of NT5DC4 was present in all six D-T2DM patients, and in addition, the PARP1 gene, which is closely related to T2DM, was mutated in three D-T2DM patients.

**Table 4:** The top 15 shared mutated genes and their annotation results

Priority	CHROM	POS	ID	GeneName	ExonicFunc	Patient shared number	Normal shared number
H	2	113479751		NT5DC4		6	0
H	2	113481035		NT5DC4	nonframeshift deletion	6	0
H	2	113483863	rs368642527	NT5DC4	missense SNV	6	0
H	1	201177415	rs139658488	IGFN1	missense SNV	4	0
H	1	201190586	rs565007693	IGFN1	missense SNV	4	0
H	1	55252706	rs201017388	TTC22	missense SNV	3	0
H	1	55252712	rs370158426	TTC22	missense SNV	3	0
H	1	55252757		TTC22		3	0
L	1	100661986	rs760164623	DBT		3	0
L	1	100661987		DBT		3	0
H +	1	226555174	rs565966803	PARP1		3	0
H +	1	226567629		PARP1	missense SNV	3	0
H +	1	226576415	rs139232092	PARP1	missense SNV	3	0
H +	1	226595617	rs201256399	PARP1	missense SNV	3	0
H	10	73464873	rs535416598	CDH23	missense SNV	3	0

### GO and KEGG pathway enrichment analysis of the mutated sites

**Fig. 2:**
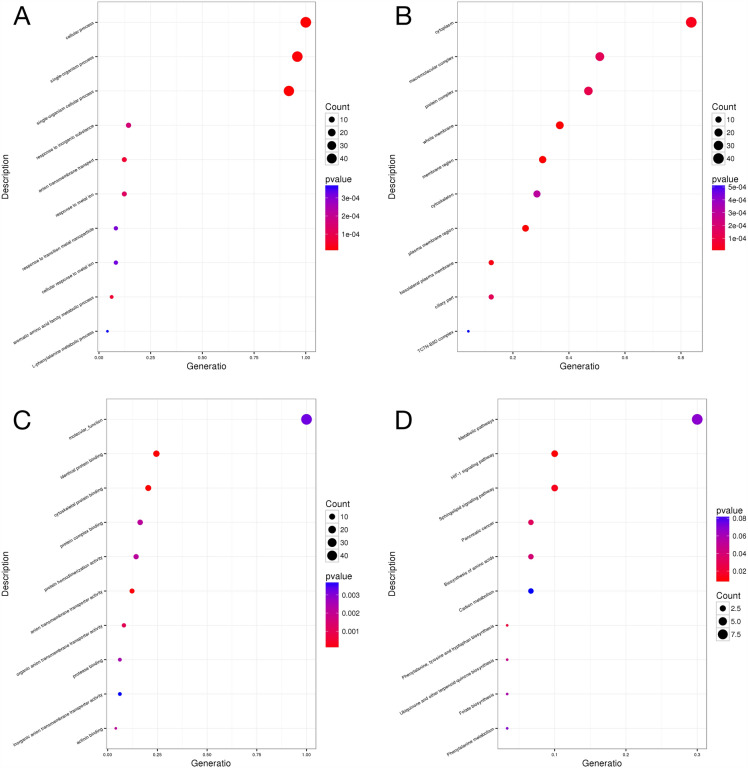
Scatter plot of the GO and KEGG pathway enrichment analysis of mutated sites. **A** GO category of BP. **B** GO category of CC. **C** GO category of MF. **D** KEGG pathway

Since different genes usually cooperate to perform their biological functions, especially in a complex disease like T2DM. To reveal the biological functions of the mutated genes, gene ontology (GO) analysis was performed to classify genes according to their biological functions. GO analysis enriched the mutated genes in three different categories, including biological process (BP), cellular component (CC), and molecular function (MF). In our results, the total number of significantly enriched BP, CC, and MF were 422, 89, and 81 (P < 0.05), respectively. As well, the most enriched term in BP, CC, and MF was cellular process (GO: 0098656), cytoplasm (GO: 0005737), and molecular function (GO: 0003674), respectively (Fig. 2A, B, C).

In addition, to further investigate the biological functions of the mutated genes in D-T2DM patients, we performed pathway enrichment analysis based on the KEGG database. The results showed that a total of 6 pathways were enriched significantly (P < 0.05). The scatterplot in Fig. 2D demonstrates the top 10 enriched pathways. Among them, the HIF-1 signaling pathway (hsa04066) and sphingolipid signaling pathway (hsa04071) are related to T2DM and mitochondrial function.

### Gene-disease phenotype correlation analysis

**Fig. 3:**
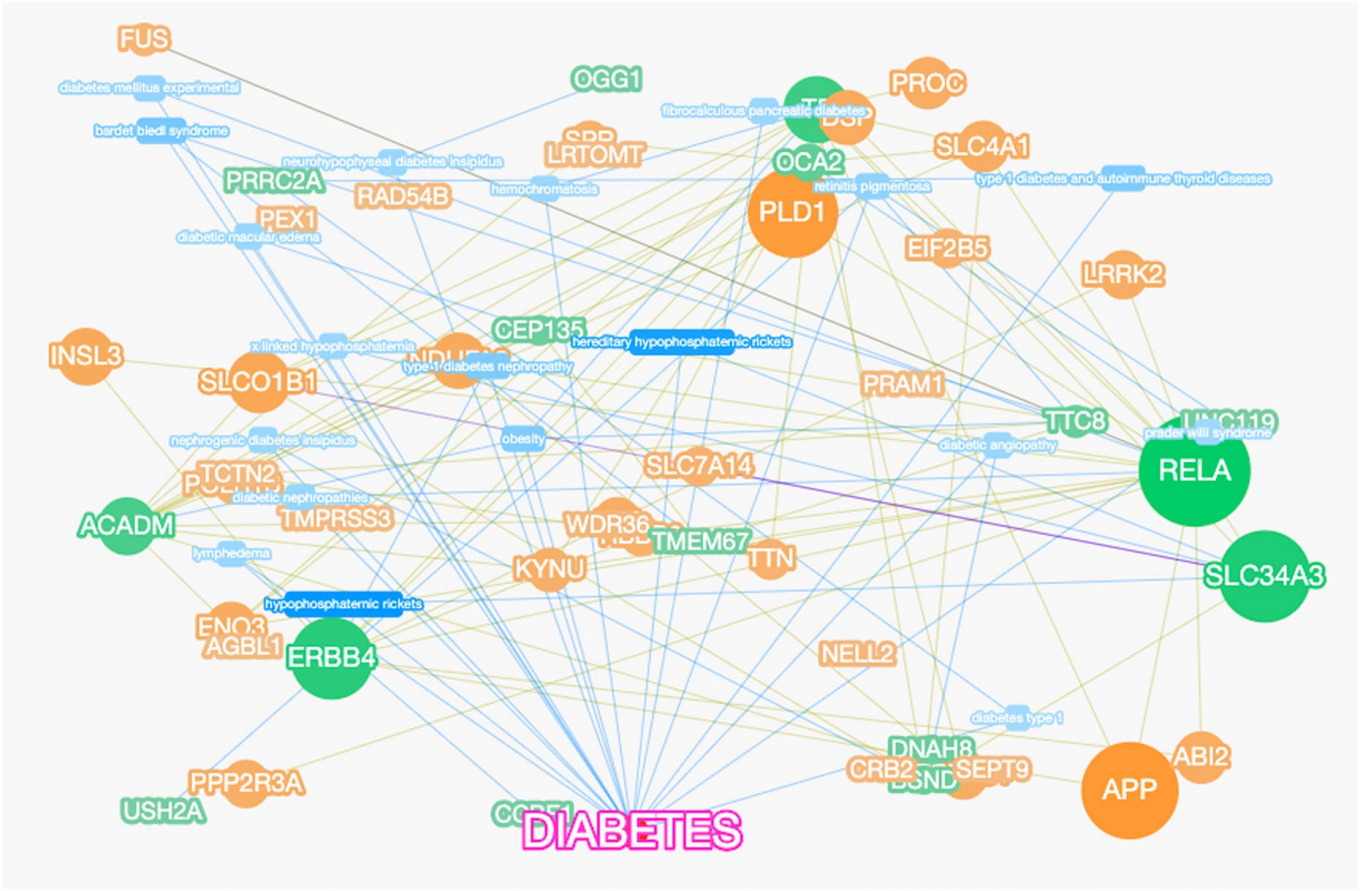
Gene-phenotype-disease association network. The circle size for each of the indicated genes represents the strength of the correlation with the disease. Nodes with a green color represent genes related to diabetes according to existing reports or databases; nodes with an orange color represent genes related to green color genes according to various associations

**Fig. 4:**
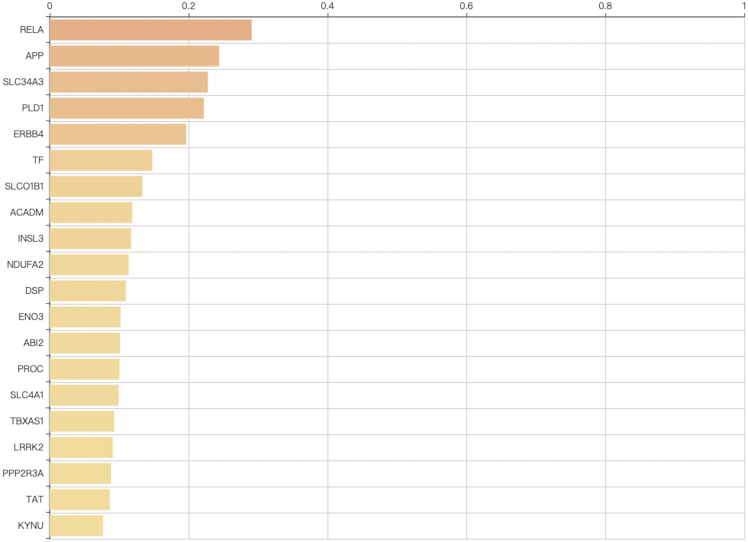
The top 20 genes in the network, which were ranked by association degree

Based on the DisGeNte database V5.0 (a database of gene-disease associations with 561,119 gene-disease and 135,588 mutation-disease association records), we studied the associations between T2DM and our sequencing results. The results showed that in our sequencing results, there were 23,230 genes associated with the development of T2DM. Next, we constructed the gene-phenotype-D-T2DM interaction network based on the first 49 genes of the candidate genes (Fig. 3). Then, we subjected the candidate genes to Phenolyzer Software to generate a ranked gene list. As illustrated in Fig. 4, the higher the rank, the higher the correlation between candidate genes and T2DM, and the top two genes with the highest rank were RELA and APP.

### Protein-protein interaction analysis

**Fig. 5:**
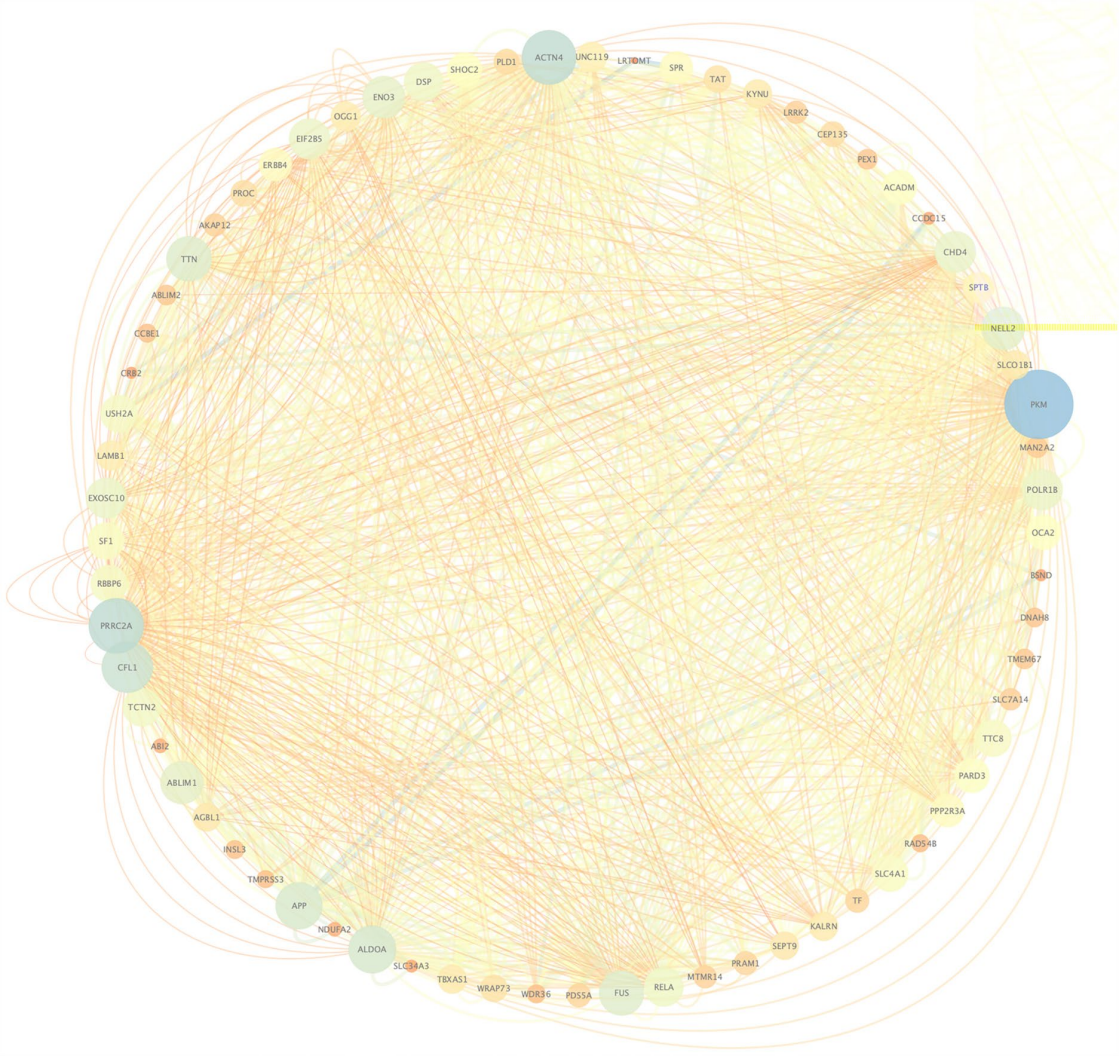
Protein–protein interaction network of the shared mutated genes

To further explore potential interactions among the shared mutated genes, we performed protein functional interaction network analysis using the online software GeneMania [15]. It includes protein–protein, protein-DNA-genetic interactions, pathways, reactions, gene-protein expression data, protein domains-phenotypic screening profiles. Afterwards, the protein–protein interaction network was constructed by Cytoscape software (Fig. 5).

### DNA and RNA joint analysis results

#### Screening of the differentially expressed consensus mutated genes in D-T2DM patients

**Fig. 6:**
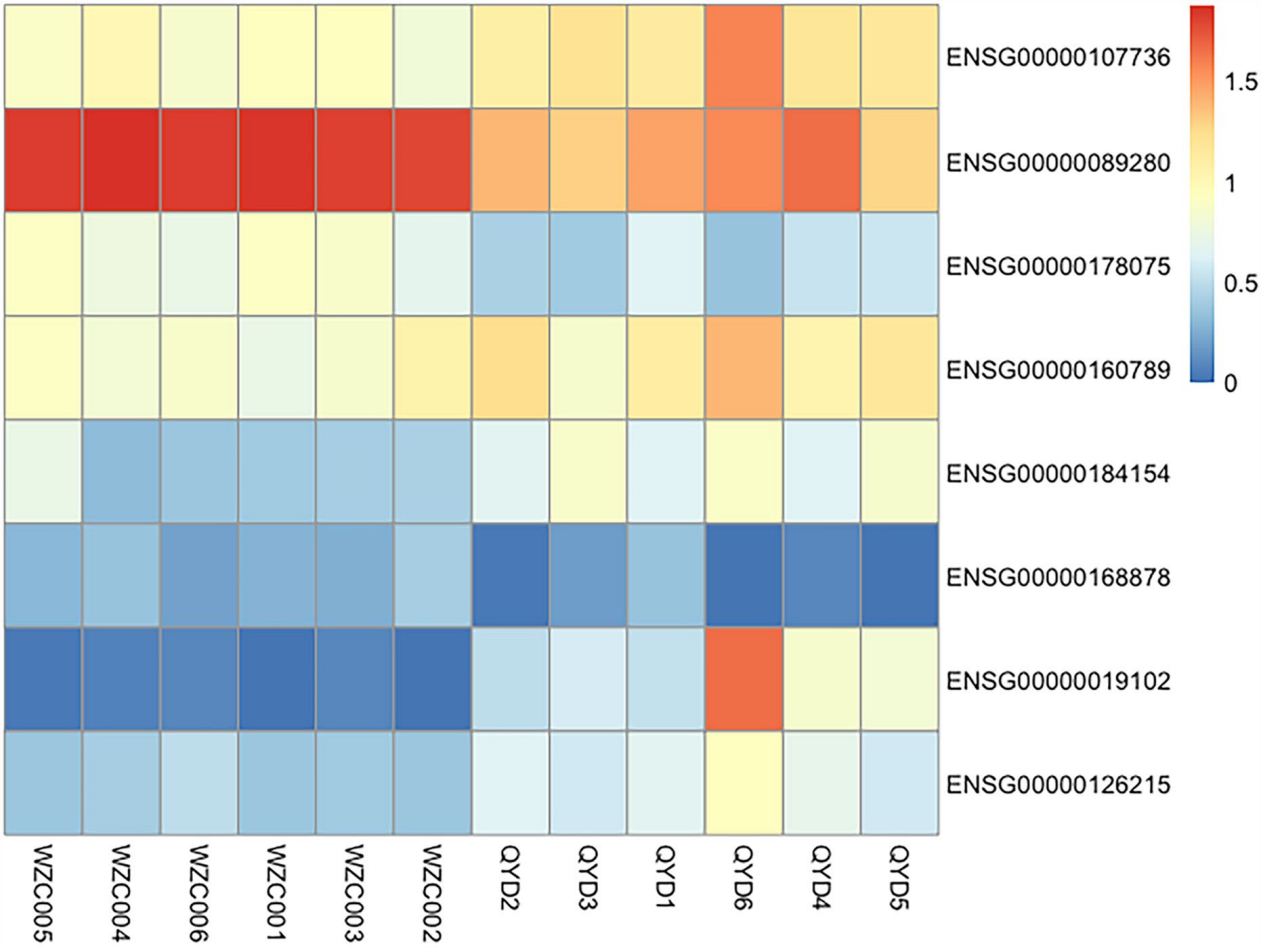
Clustering heatmap of differentially expressed consensus mutated genes

We thought that if a gene is a shared mutated gene in D-T2DM patients and significantly differentially express in D-T2DM patients compared with normal people, this gene may be critical for the development of D-T2DM. Therefore, we analyzed the relative expression of the shared mutated genes at the RNA level. Differential gene expression was assessed using the Cuffdiff software. Then, based on the sequencing depth and gene length for the reads count, FPKM was chosen to express the expression values of genes. The results were shown in Fig. 6 and Table 5: eight shared mutate genes were screened with significant differential expression. Among them, the CDH23 gene mutated in three patients, the rest of the mutated genes were shared with two patients. Moreover, CHD23, LMNA, LRTOMT, VSIG2, and XRCC3 were up-regulated, well FUS, GRAMD1C, and SFTPB were down-regulated in D-T2DM patients compared with normal individuals.

**Table 5:** Mutant genes and differentially expressed genes in D-T2DM patients

Gene name	Number of patients	Corresponding transcript name	Log2 Fold Change	P value of DE RNA	q value of DE RNA
CDH23	3	ENSG00000107736	1.33058	0.00415	0.0562885
FUS	2	ENSG00000089280	− 1.24573	5.00E−05	0.00240707
GRAMD1C	2	ENSG00000178075	− 1.42239	0.00085	0.0200327
LMNA	2	ENSG00000160789	1.04216	5.00E−05	0.00240707
LRTOMT	2	ENSG00000184154	1.40101	0.0154	0.126948
SFTPB	2	ENSG00000168878	− 1.52488	0.0496	0.270932
VSIG2	2	ENSG00000019102	6.72329	0.0011	0.0235009
XRCC3	2	ENSG00000126215	1.42051	0.0026	0.0422863

#### GO and KEGG pathway analysis of the differentially expressed consensus mutant genes in D-T2DM patients

**Fig. 7:**
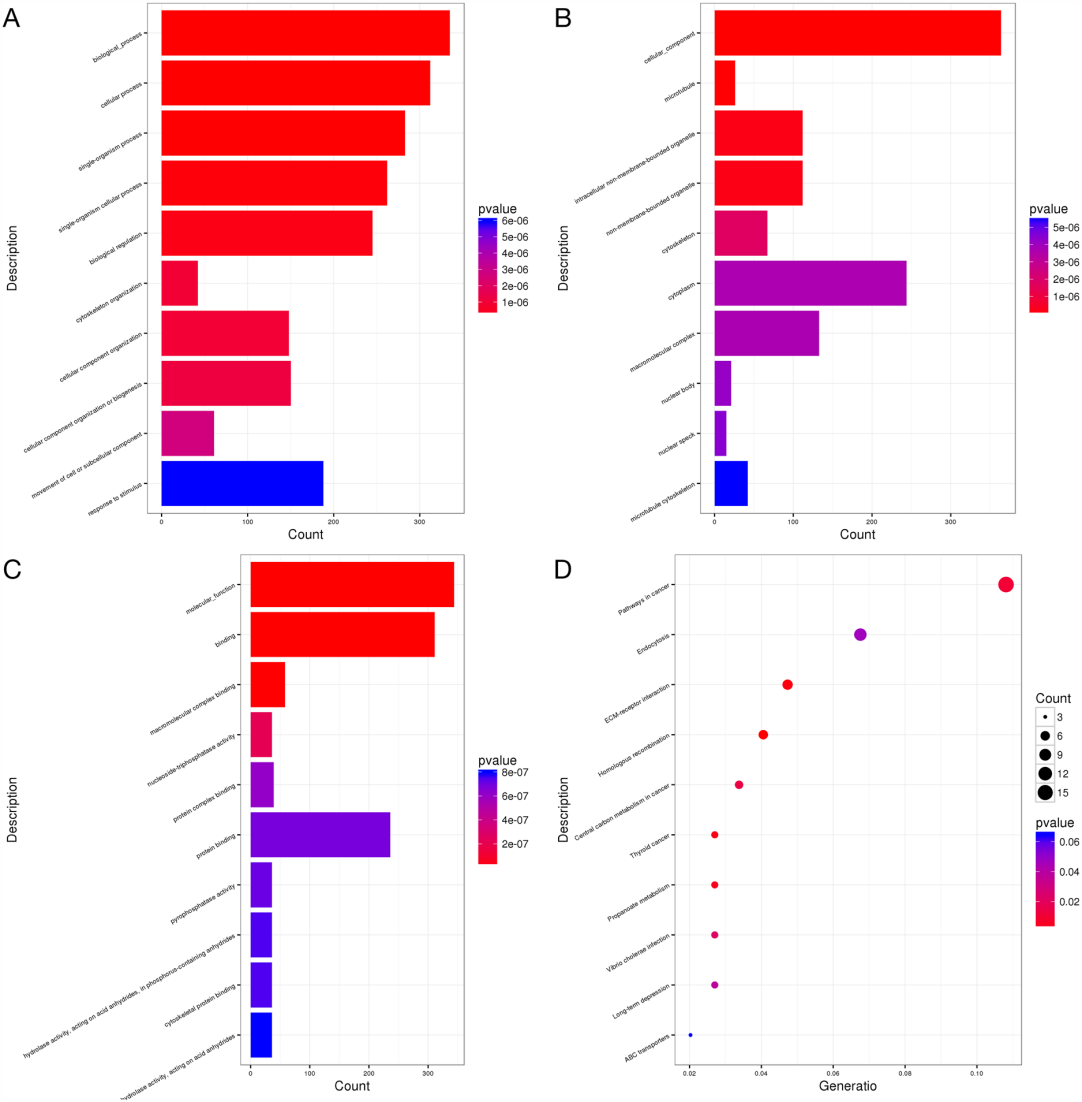
The histogram of GO and the scatterplot of the KEGG pathway analysis. **A** Biological process. **B** Cellular Component. **C** Molecular Function. **D** KEGG pathway

In order to identify a potential biological function for the key genes which were shared mutated genes in DNA level and differentially expressed in RNA level, GO and KEGG pathway analysis were proceed on these key genes. As shown in Fig. 7, the most significantly enriched BP, CC, and MF entry was the biological process, cellular component, and molecular function, respectively. The pathways in cancer, endocytosis, and ECM-receptor interaction were significantly enriched in the KEGG analysis.

#### Drug prediction based on the interaction network of herbs and the differentially expressed consensus mutant genes

**Fig. 8:**
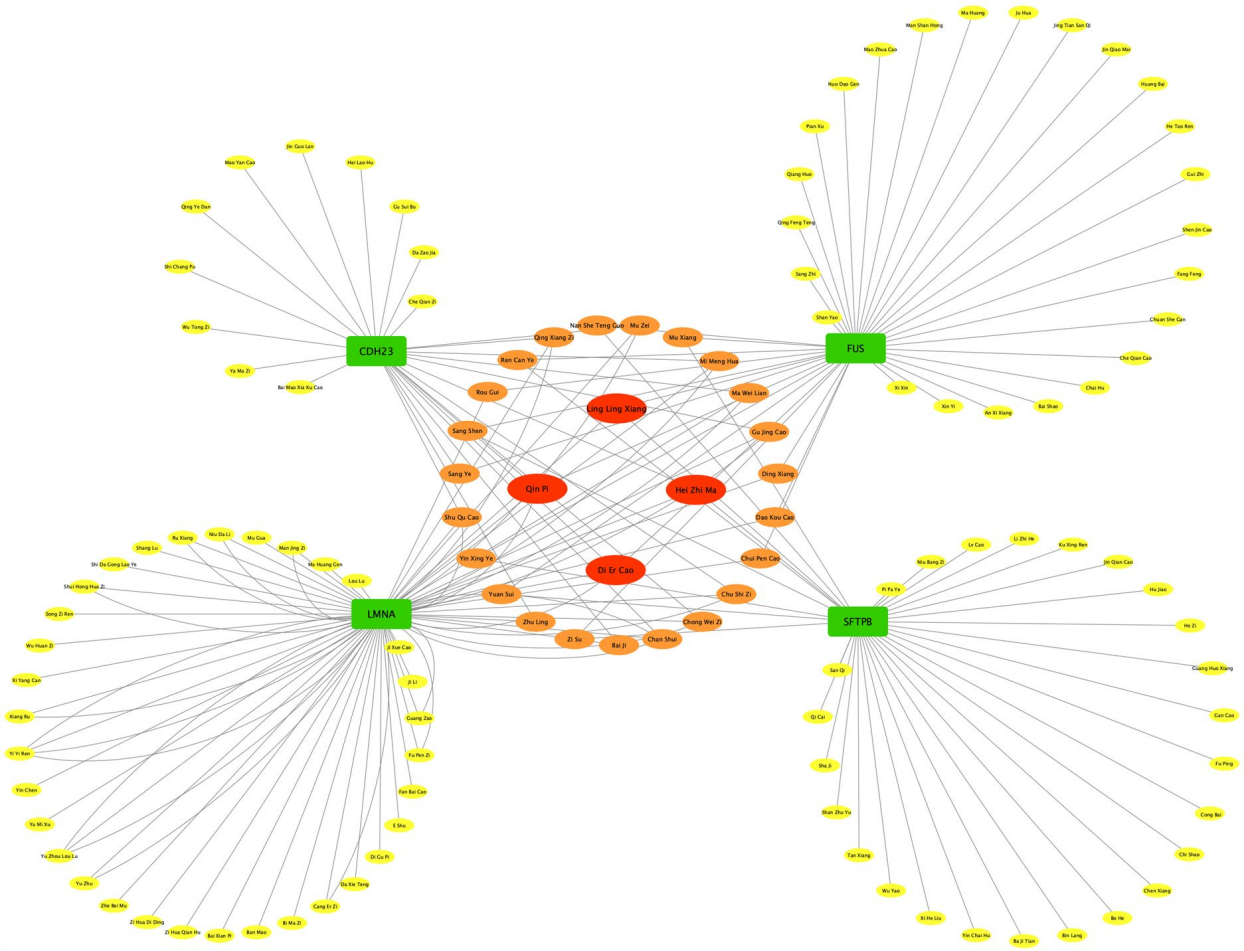
Herb-gene interaction network. Green squares represent the differentially expressed consensus mutant genes; Circles represent the predicted herbs; Different colors represent the different number of herbs associated with the node, yellow nodes associated with one herb, orange nodes associated with two herbs, red nodes associated with three herbs

To further predict herbs that might act on the differentially expressed consensus mutant genes, we constructed an interaction network of these genes with herbs through the SymMap database. As shown in Fig. 8, four genes could be predicted with their associated herbs through the database. A total of 26, 39, 52, and 34 herbs were associated with CDH23, FUS, LMNA, and SFTPB, respectively. Among these herbs, 23 were related to two of the four genes, and four were related to three of the four genes. These four herbs were Qin Pi (*Cortex Fraxini,* bark of Largeleaf Chinese Ash), Ling Ling Xiang (Lysimachiae Foenigraeci Herba), Hei Zhi Ma (*Semen Sesami Nigrum*, Black Sesame), and Di Er Cao (*Herba Hyperici Japonici*, all-grass of Japanese St. Johnswort).

## Discussion

In our work, we subjected 12 individuals (six D-T2DM patients and six normal individuals) to whole-exome sequencing. After comparison with the existing database, the mutation site was screened out. Then we classified the pathogenicity of these mutations, screened the mutant genes shared in patients, and performed a series of bioinformatics analyses like GO, KEGG, and gene-disease phenotypic correlation analysis. Next, we combined alteration of genetic information of DNA level with RNA level to understand the pathogenesis of D-T2DM further and provide new targets for D-T2DM diagnosis and treatment.

After sample preparation, sequencing procedure and analysis, consensus mutations were screened out. There was a total of 235 consensus mutations. Among them, NT5DC4 has mutations in all 6 D-T2DM patients. Although there have been no studies showing the relationship between NT5DC4 and T2DM, we speculated that NT5DC4 might play a role in the pathogenesis of D-T2DM. In addition, PARP1, poly(ADP-ribose) polymerase 1, is related to immune response, inflammation, and infection responses in diabetes [16]. Studies have shown that PARP1 regulates MMP-9 expression to maintain mitochondrial homeostasis through manipulating the binding of NF-kB/AP-1 at the MMP-9 promoter [17]. Nuclear PARP1 rapidly triggers mitochondrial dysfunction, and the inhibition of PARP can protect mitochondria and reduce ROS production via PARP-1-ATF4-MKP-1-MAPK retrograde pathway [16, 18]. Mitochondria are essential organelles that provide energy in the form of adenosine triphosphate (ATP), including energy needed for muscle contraction, nerve impulses and the synthesis of all complex molecules in the body [19]. Thus, mitochondrial dysfunction will result in fatigue. In Traditional Chinese Medicine, the lack of “Qi” causes fatigue, a hallmark symptom of mitochondrial disease [20]. In addition, impaired mitochondrial function plays an important role in the development of diabetes and insulin resistance [21]. Therefore, the mechanism of D-T2DM may be related to mitochondrial dysfunction. In this study, we identified three missense mutations of PARP1, including G1537A in exon 10, C659T in exon 5, and C14G in exon 1. Hence, it could conceivably be hypothesized that the mutation of PARP1 is possibly associated with the mitochondrial dysfunction in D-T2DM patients.

To further reveal the metabolic pathways associated with the consensus mutations of D-T2DM patients, we performed a KEGG pathway enrichment analysis. The results showed that there was significant enrichment of 6 pathways. Based on previous studies, we found that some of these pathways may be associated with the pathogenesis of D-T2DM, including the metabolic pathway, HIF-1 signaling pathway, and sphingolipid signaling pathway. HIF-1 signaling pathway has well-established roles in insulin secretion and glucose homeostasis [22]. Previous studies established that HIF-1 regulates several critical pathways in the adaptive responses of cells to hypoxia [23]. In addition, HIF-1 could influence mitochondrial function by suppressing both the TCA cycle and respiration and controls mitochondrial biogenesis and autophacy [24]. Sphingolipids maintain the structural integrity of cell membrane and regulate multiple critical cellular processes through signal transduction and gene regulation, which are related to the occurrence and development of various diseases such as diabetes, inflammatory bowel disease, asthma, etc [25, 26]. Furthermore, sphingolipids metabolize nearby mitochondria and regulate mitochondrial structure and function [27]. The dysregulated sphingolipid metabolism leads to mitochondrial dysfunction and disease [27]. In our study, the consensus mutation gene in D-T2DM patients is significantly enriched in HIF-1 and sphingolipid signaling pathways. Consequently, we speculated that the consensus mutant gene might influence the development of D-T2DM through functioning in pathways associated with glycan metabolism, energy metabolism, and mitochondrial disease.

In disease-related research, it is indispensable to identify the correlation between candidate genes and human disease. Therefore, by comparing with the database, we constructed a gene-phenotype-D-T2DM correlation network. In the results, a total of 23 230 genes were found. In addition, we ranked these genes base on their relevance with T2DM. The results showed that the top two relevant genes were RELA (RELA proto-oncogene, NF-kB subunit) and APP (amyloid-beta precursor protein). The combination of NFKB1and RELA constitutes the most abundant form of NF-kappa-B, a ubiquitous transcription factor involved in several biological processes. The activation of NF-kappa-B is a crucial event early in the pathogenesis of diabetes [28]. In addition, NF-kappa-B is a physiological regulator of mitochondrial respiration and plays a role in metabolic adaptation in cells [29]. APP, a precursor protein of amyloid-beta (Aβ), is first cleaved by either α-secretase or β-secretase to produce CTFs 83aa (C83) or 99aa (C99) long, respectively. Then, C83 and C99 are cleaved by PS1 and PS2 to produce p3 or Aβ, respectively. A recent study has reviewed that Aβ could affect insulin sensitivity, reduce glucose-dependent insulin secretion and contribute to the onset of diabetes [30]. Furthermore, mitochondria are one of the major targets that Aβ oligomers negatively impact, including the impairment of fast transport and fragmentation [31]. In addition, C99, the intermediate of Aβ, was a driver of mitochondrial dysfunction in Alzheimer's disease and mediated by the loss of sphingolipid homeostasis [32]. Therefore, since RELA and APP were closely related to T2DM and mitochondrial dysfunction, we believe that RELA and APP may serve as a clinical predictor of the diagnosis in patients with D-T2DM.

D-T2DM is a multifactor disease caused by a combination of environmental and genetic factors. In recent years, with the development of high-throughput sequencing technology, significant breakthroughs in the study on the occurrence and development of D-T2DM have been achieved. But a single-omic approach can only reveal the changes at an individual level. Accordingly, to deepen our understanding of the development mechanisms of D-T2DM, our efforts should focus on integrating multi-omics datasets. The sequencing of the genome contains important information regarding the underlying genetic variations in D-T2DM. The transcriptome sequencing using high-throughput methods enables studies on gene expression of D-T2DM patients and links genome to proteome. Consequently, we combined the sequencing of DNA and RNA level to establish the relationship between the information of genomic mutation and the changes of transcriptome expression.

After the combined analysis, we screened out eight genes altered on both DNA and RNA levels. Among these genes, FUS (FUS RNA binding protein) could interact with HSP60, a mitochondrial chaperonin, to promote mitochondrial damage [33]. The previous study has demonstrated that mitochondrial ATP production is impaired in FUS-expressing cells [34]. In our research, the FUS gene showed missense mutation at rs201533156 sites in the exonic region and significantly down-regulated (log2foldchange = − 1.24573) in D-T2DM patients compared with normal individuals. Lamin A/C (LMNA) plays a role in intracellular redox homeostasis. A study by Tom Sieprath et al. demonstrated that persistent LMNA depletion elevates reactive oxygen species (ROS) levels [35]. ROS, as prime modulators of cellular dysfunction contribution to disease pathophysiology, can act in the mitochondrial energy metabolism and the regulation of metabolic/inflammatory diseases such as diabetes [36]. In this study, we found that the LMNA gene mutated at rs200917748 and significantly up-regulated in mRNA expression levels (log2foldchange = 1.04216) in D-T2DM patients compared with normal individuals. Thus, we hypothesized that the FUS and LMNA genes might play a role in the pathogenesis of D-T2DM. Furthermore, the other six screened genes are also differentially expressed genes, and all have common mutations in D-T2DM patients. Although no previous studies have provided information on the relationship between these genes and T2DM or fatigue, we believe that these genes may have a role in the pathogenesis of D-T2DM because they changed at both the DNA and RNA levels.

To guide subsequent drug studies, we further predicted Chinese herbs associated with the key genes through the database. In total, four herbs were more correlated with differentially expressed shared mutations in the interaction network. Among them, Qin Pi is known to possess anti-inflammatory and anti-oxidative stress effects [37, 38]. In the previous researches by Prabakaran et al., esculetin, one of the main active ingredients in Qin Pi, can exert antioxidant and anti-hyperglycemic capacity in diabetic rats [39, 40]. Hei Zhi Ma is rich in nutrients and has the effect of tonifying the liver and kidney and replenishing vital and blood in TCM theory [41]. Many recent studies have shown that Hei Zhi Ma extract has the ability to improve insulin resistance and reduce blood glucose [42, 43]. In addition, according to the Chinese Pharmacopoeia, Ling Ling Xiang and Di Er Cao can be used to treat fatigue [41]. Accordingly, these herbs were likely to be able to exert the therapeutic effect on D-T2DM. Further experiments need to be done to investigate the detailed mechanisms.

## Conclusions

Taken together, this study set out to gain a greater understanding of the mechanisms of occurrence and development of D-T2DM. After whole-exome sequencing on peripheral blood of D-T2DM patients, the consensus mutations were screened out and analyzed by a series of bioinformatics analyses. Then, we combined the results of WES and transcriptome sequencing, and eight genes, including FUS and LMNA, were found that changed at both the DNA and RNA levels. Subsequently, we predicted the herbs that might play a therapeutic role in D-T2DM through the database. However, the pathological mechanism of D-T2DM is complex and multifactorial. The peripheral blood selected as the subject in this study can only partially explain the mechanism of D-T2DM. Therefore, in the future, we intend to investigate the muscle, islet and adipose tissues of D-T2DM patients to further elucidate the developmental mechanism of D-T2DM. In summary, our findings have important implications for understanding the mechanisms of D-T2DM and provide potential targets for D-T2DM diagnosis and treatment.

## Supplementary Information

**Additional file 1: Table S1.** summary of the quality of data. **Table S2.** Comparison rate and coverage. **Table S3.** number of InDel in different regions of the genome. **Table S4.** number of different types of InDel on the coding area. **Table S5.** Annotation of mutation cite.

## Data Availability

The datasets used and/or analysed during the current study are available from the corresponding author on reasonable request.

## Abbreviations

Aβ: Amyloid-beta
ACMG: The American Society of Medical Genetics and Genomics
ATP: Adenosine triphosphate
BP: Biological process
C83: CTFs 83aa
C99: CTFs 99aa
CC: Cellular component
CNV: Copy number variations
D-T2DM: Type 2 diabetes mellitus patients with fatigue
GO: Gene Ontology
InDel: Insertion and deletion
KEGG: Kyoto encyclopedia of genes and genomes
MF: Molecular function
SNP: Single nucleotide polymorphisms
T2DM: Type 2 diabetes mellitus
WES: Whole-exome sequencing technology
